# How Does Environmental Information Disclosure Affect Pollution Emissions: Firm-Level Evidence from China

**DOI:** 10.3390/ijerph191912763

**Published:** 2022-10-06

**Authors:** Xiangyang Yang, Zheng Zhang, Siqi Rao, Bei Liu, Yueyue Li

**Affiliations:** 1 School of International Economics and Trade, Nanjing University of Finance and Economics, Nanjing 210023, China; 2 Institute of Food and Strategic Reserves, Nanjing University of Finance and Economics, Nanjing 210023, China; 3 School of Business, Hunan Normal University, Changsha 410081, China; 4 School of Management, Nanjing University of Posts and Telecommunications, Nanjing 210003, China

**Keywords:** environmental information announcement, front-end of technological innovation, difference-in-differences method, end-of-end environmental governance

## Abstract

This paper uses the environmental information announcement system as a quasi-natural experiment, cleaning China’s Industrial Enterprise Pollution Database, a unique and comprehensive firm-level database, and merges it with China’s Industrial Enterprise Database. Then, we use the difference-in-differences model to test the effect of environmental information announcements on firm pollution emissions and the transmission mechanism. The empirical results found that environmental information announcement has a significant environmental performance improvement effect. That is, environmental information announcements can significantly reduce pollution emissions. Moreover, the effects of environmental information announcement differ significantly under different regions, city levels, and environmental regulatory intensities. Specifically, in the eastern region, first-class cities, and regions with higher environmental regulations, the emission reduction effects of enterprises are more obvious. Further transmission mechanism test results show that environmental information disclosure has a dual emission reduction mechanism of internal driving and external pressure. Front-end of technological innovation and end-of-end environmental governance are important manifestations in internal driving. Under external pressure, companies will reduce production so as to achieve the goal of reducing pollution emissions.

## 1. Introduction

Since the reform and opening-up, China’s economy has had high growth for more than 40 years, and it is also inevitably bringing large emissions of pollution and a sharp decline in environmental quality. Data from the 2018 China Ecological and Environmental Status Bulletin showed that among the 338 prefecture-level and above cities that year, only 121 cities could meet the urban ambient air quality standards, accounting for 35.8% of the total number of cities. In addition, the environmental performance report released by Yale University in 2018 pointed out that China’s comprehensive environmental index ranked 120th among all 180 countries participating in the election, and the air quality index ranked 177th. Obviously, China’s environmental governance has become a pressing issue [1], for that environmental pollution will not only seriously affect residents’ quality of life and people’s health, but also bring a negative impact on China’s sustainable economic development. In recent years, energy consumption and environmental quality issues have attracted more and more attention [2]. In this context, the report of the 19th CPC National Congress clearly pointed out that ecological and environmental protection has a long way to go. It is necessary to continue to implement air pollution prevention and control actions, win the battle for blue sky, and establish and practice the concept that lucid waters and lush mountains are invaluable assets. Under the circumstance of the new normal economy, China’s economy has changed from a stage of rapid growth to a stage of high-quality growth. At present, China’s economy is in a critical period of high-quality economic development [3]. In this process, reducing pollution emissions has become an indispensable part of high-quality economic development, and it is also an urgent problem to be solved. 

Environmental problems are considered to be an important proposition related to human survival. Despite China’s economic development is advancing and has become the world’s second-largest economy, the ensuing environmental pollution problems have laid hidden dangers for China’s sustainable development. China has successively promulgated a series of environmental regulatory measures aimed at reducing pollution emissions. In fact, as early as 1983, environmental protection has been listed by The State Council as a basic state policy of China’s economic and social development. Since the end of the 20th century, a series of laws related to environmental regulations successively promulgated by the Chinese government has conspicuously improved China’s environmental problems [4]. In this context, regulations of the Environmental Protection Law of the People’s Republic of China, The Law of the People’s Republic of China on the Prevention and Control of Atmospheric Pollution, Ambient Air Quality Standards, etc., had also been implemented one after another, among which The Law of the People’s Republic of China on the Prevention and Control of Atmospheric Pollution was called the “strictest” environmental protection law in history. The promulgation of this series of laws shows that the state attaches great importance to environmental protection. The increasingly stringent environmental regulations also demonstrate the country’s determination to deal with environmental pollution problems. In order to further reduce pollution, propel local governments environmental protection and promote the green production of polluting enterprises, the Ministry of Ecology and Environmental Protection of the PRC officially issued the “Measures for the Announcement of Environmental Information (for Trial Implementation)” on 8 February 2007 (hereinafter referred to as the “files”), and began to implement it in May 2008. The “files” aim at the promotion of the release and sharing of environmental information in local cities in China and put forward specific requirements for environmental information announcement from both the local government and the enterprise.

Under the framework of environmental legislation, environmental information disclosure, as an effective way of government and public supervision, can significantly promote the green transformation of enterprises through external pressure and image reputation effect, which is closely related to enterprise environmental performance. As an important institutional arrangement for enterprises to actively assume social responsibility, can environmental information disclosure effectively promote low-carbon development of enterprises? Under the current background of “carbon peak” and “carbon neutrality”, it is of great practical significance to explore the impact of environmental information disclosure on enterprise emission reduction. In view of this, this article starts with the environmental information announcement system to examine the impact of environmental information announcement on corporate emission reduction. Cleaning China’s Industrial Enterprise Pollution Database and merging it with China’s Industrial Enterprise Database in the period of 1998–2012, with the combination of the “Environmental Information Disclosure Measures”, which was officially implemented in 2008, this article uses the difference-in-difference (DID) model to assess the impact of the environmental information announcement system on the firm emission reduction. The marginal contributions of this paper are as follows: (1) We fetch up the blank that although there is much literature exploring the effect of environmental regulations on pollution emissions, few studies are concerned about the effect of environmental information announcement system, which is kind of "information transmission" regulations, on pollution emissions. (2) Differing from existing literature that mainly concentrates on the macro prefecture-level city level, this paper, considering that enterprises which are the main body of pollution discharge and prevention play a vital role in China’s environmental protection, explores the pollution emissions impact of environmental information announcement from the micro-enterprise level, which expands the research ideas and also provides micro-level evidence on the implementation of environmental information disclosure systems. (3) We analyze the transmission mechanism of the environmental information announcement system on the enterprise’s emission reduction behavior and use the empirical method to verify it. It provides guidance for how an environmental information announcement system can better promote the enterprise’s emission reduction.

The remainder of the paper is arranged as follows: the second part is the method and design, explaining the empirical method and the composition of the data used in this article; the third part conducts empirical research on the research theme of this article and analyzes the corresponding results; the fourth part studies the transmission mechanism; the fifth part provides the research conclusions and policy recommendations.

## 2. Literature Review

Existing literature has mostly focused on the effect of pollution emissions brought by environmental regulations, which in the perspective of executant executor can be divided into formal environmental regulations led by the government and informal environmental regulations relying on the participation of the masses and society. There are two different opinions about the impact of formal environmental regulations on pollution emissions in academics: Some scholars hold the opinion that formal environmental regulations can effectively reduce pollution emissions. Zhang et al. (2019) argue that the government’s smog control policies help improve the atmospheric environment and improve the quality of economic development [5]. Environmental tax, as a concrete measure of environmental regulations, is an important means to promote enterprise pollution reduction [6], has also attracted the attention of many scholars. Environmental taxes can encourage companies to increase the treatment of pollutants and reduce pollution levels by reducing pollution emissions per unit of output [7]. Meanwhile, in order to reduce the cost of pollution reduction in enterprises, many countries have begun to implement environmental regulation policies of pollution reduction subsidies. Aghion et al. (2016) indicate that taxes on polluting companies and subsidies for clean companies can reduce the level of environmental pollution [8].

In addition, some scholars suggest that because of the problems of “government-enterprise collusion”, “formalism”, and “one size fits all” in Chinese society, formal environmental regulations not only have no significant impact on pollution reduction, but even promote the discharge of pollutants [9,10]. Blackman and Kildegaard (2010) explore the pollution effects of government environmental regulations in Mexico, which the study shows exacerbate pollution emissions [11]. Shibli and Markandya (1995) found that China’s pollution discharge fee system has not been effectively implemented, but more local financing means and cannot reduce pollution emissions [12]. Cheng et al. (2017) used dynamic spatial panel models to find that the emission reduction effect of market-based environmental regulations is limited, while some other scholars argue that the inefficiency of environmental governance should not be entirely attributed to local government’s inaction or government-enterprise collusion [13]. Because under the circumstance of increasingly stringent environmental regulations in the region, enterprises can not only reduce pollutant emissions through technological innovation, but also reduce environmental governance costs by relocating the site to an area with weaker environmental regulations, which is commonly referred to as the pollution refuge effect.

The development of information asymmetry theory enriches the research perspective of environmental regulation, and more and more scholars hold the opinion that informal environmental regulation can also effectively curb firm pollution emissions. The concept of informal regulations was first proposed by Pargal and Wheeler (1996), arguing that where formal regulations are weak or perceived to be insufficient, communities may informally promote pollution reduction through negotiations with local polluters [14]. Many scholars aboard have begun to pay attention to the impact of informal environmental regulations on firm pollution reduction. Additionally, some of them regard the publication of environmental pollution and improving the environmental performance of listed companies as a way of informal environmental regulations to studying how the capital market responds to the enterprises with environment information announcements. Using the difference-in-difference (DID) method, García et al. (2009) treat firms participating in the Indonesian Public Disclosure Project as the treatment group and other firms as the control group [15]. The empirical test found that environmental information announcements can alleviate environmental pollution. Langpap and Shimshack (2010) point out that public law enforcement and public supervision contribute to the management of water pollution [16]. Further, Kathuria (2007) argues that informal environmental regulation in developing countries has inevitable limitations, especially for small and medium-sized polluting enterprises [17]. Meanwhile, informal environmental regulations in China have also attracted the attention of part of scholars. Based on the sample of 85 cities and towns in China, with the informal environmental regulations level measured by the number of residents’ environmental complaints, Wang and Di (2002) find that residents’ environmental pollution complaints can help improve the level of environmental pollution control in local governments [18]. Similarly, based on the water quality data from 106 monitoring points in India, with informal environmental regulations measured in terms of the voter turnout in parliamentary elections, Goldar and Banerjee (2004) explore and find that there is a positive correlation between the polled percentage and regional water quality [19].

Although there has been a lot of literature on the impact of environmental regulation on pollution emissions, most studies use comprehensive environmental indicators or pollution-related indicators to measure environmental regulation [20,21], which inevitably creates endogenous problems and obscures emission reduction effects of environmental regulations. Therefore, abundant literature begins to use proxy indicators, represented by pollution tax rates and pollution control costs. However, there are some defects in proxy indicators of the pollution tax rate, which not only depends on the intensity of environmental regulations, but also is closely related to many other factors such as the local economic development stage and enterprise pollution behavior, so it is also not advisable to completely attribute the change of pollution tax rate to the intensity of environmental regulations. However, the official implementation of the environmental information announcement in 2008 provides a new perspective for investigating the emission reduction effects of environmental regulations. As an excellent exogenous shock, the environmental information announcement provides this study of the impact of environmental regulations on firm emission reductions with a quasi-natural experiment, where the usage of DID method helps us to effectively solve endogenous problems, and thus to obtain an accurate assessment of how environmental information announcement affects pollution emissions.

## 3. Method and Design

### 3.1. Benchmark Model

**Figure 1:**
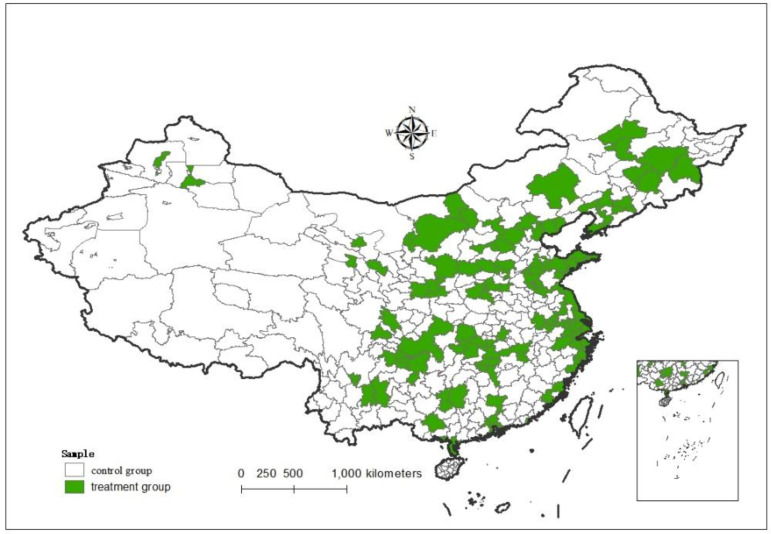
Spatial distribution of environmental information disclosure pilot areas in China.

The DID method, also known as the double-difference method, can effectively solve the endogenous problem under the assumption. In recent years, it has been widely used in many fields such as economics and management and has become the most widely used causal inference method [22,23,24]. Based on the above analysis of advantages, the environmental information disclosure system, as a perfect exogenous impact, provides a quasi-natural experiment for this paper to study the impact of environmental regulation on enterprise emission reduction. The Institute of Public and Environmental Affairs (IPE) and the Natural Resources Conservation Commission (NRDC) have published the “Pollution Information Transparency Index (PITI)” of 113 environmentally friendly cities that have implemented environmental information announcements year by year since 2008. The index, quantitatively being evaluated from five aspects: daily supervision, self-monitoring, report response, emission data and environmental impact assessment information, can effectively reflect the intensity of environmental information announcement, while other prefecture-level cities that have not implemented environmental information announcement systems need not publish relevant environmental bulletins and firm pollution emissions, which provides conditions for this article to construct a “quasi-natural experiment” and use the DID method to explore the impact of environmental information disclosure systems on pollution emissions. Among them, 113 cities implementing information announcement were treated as the treatment group, and the other cities not implementing information announcement were treated as the control group, as shown in Figure 1. Through the comparison between these two groups, we assess the effect of the environmental information announcement system on pollution discharge. The model is as follows:

(1) $$P_{it}=\alpha_{0}+\beta_{t}\left(treat_{i}\times post_{t}\right)+\beta_{i}X_{it}+\delta_{i}+\mu_{t}+\varepsilon_{it}$$

where the dependent variable $P_{it}$ represents pollutant emissions of the company *i* in year *t*, $treat_{i}$ is defined as the city grouping variable that takes 1 if the city is listed in the “files”, otherwise, 0; $post_{t}$ is the time grouping variable, that is, the dummy variable for the implementation of the policy, equaling 1 for years after 2008 and equaling 0 otherwise for the reason that the policy was formally implemented from 2008; $X_{it}$ is a set of control variables.

In Formula (1), $\alpha_{0}$ is the intercept term that does not vary with the individual, $\beta_{t}$ is the estimated coefficient of each explanatory independent variable, $\delta_{i}$ is the time-fixed effect, $\mu_{i}$ is the company-fixed effect, and $\varepsilon_{it}$ is the random error term.

### 3.2. Index Design and Data Source

*Dependent variable:* Pollution emissions. This paper takes the logarithm of SO_2_ emissions (*lnSO*) as an indicator to measure pollution emissions for the reasons as follows: First, a large amount of SO_2_ is produced in the production process of Chinese industrial enterprises owing to their main energy use is coal, and simultaneously SO_2_ is also one of the most important indicators of atmosphere pollution; Second, SO_2_ is easily perceivable by the public, for which it have always been listed as the main pollutant in emission reduction policies formulated by China over the years.

*Core independent variable*: Whether the prefecture-level city was listed as an environmental information disclosure city (PITI) in 2008 and beyond. In this paper, we define the 113 prefecture-level cities released in 2008 for environmental information announcement as the treatment group, and the other cities are the control group. For cities in the treatment group, variable PITI takes 1 for years after 2008 and takes 0 otherwise, while, for cities in the control group, variable PITI always takes 0.

*Control variables*: In addition to environmental regulations and policies, there are still many factors that affect pollutant emissions in enterprises. Therefore, to reduce the error of the assessment of firm emission reduction effect of environmental information announcement system, control variables are added to the model, including enterprise size, enterprise age, enterprise revenue, and enterprise ownership type. Among them, the size of the company (*lnsca*) is characterized by the logarithm of the company’s total assets. The age of the company (*lnage*) is represented by the logarithm of the result of current year minus the year the company was founded plus 1. The revenue of the company (*lninc*) is represented by the logarithm of companies’ main business sales revenue. The type of enterprise ownership (*soe*) takes 1 for state-owned enterprises and 0 for non-state-owned enterprises.

The data used in this article mainly comes from two databases: The one is the China’s Industrial Enterprise Database from 1998 to 2012, which contains information of enterprise size, age, revenue, and ownership types used in this article, but it lacks data such as firm pollution emissions and pollution treatment equipment; The other is China Industrial Enterprise Pollution Database, which, conversely, lacks corporate finance-related information but contains detailed lists of various types of pollutant emissions of enterprise production and operation, which can be used as the dependent variables of this article. Therefore, merging these two databases can meet the needs of this paper. The concrete merging process is shown as follows: First, merging cities in the two databases by using the common variable of the company code, and by using the company name for those cannot be matched by the company code. Second, extract the information we need, respectively, from these two databases and further match with the company address. Based on all above, taking into account the impact of environmental information announcement on firm pollutant emissions can meet the needs of this research, the final research sample is obtained after additionally excluding companies without pollutant emissions, that is, SO_2_ emissions less than or equal to 0. Variable descriptive statistics are shown in Table 1.

## 4. The Effect of Environmental Information Announcements on Enterprises Pollution Emissions 

### 4.1. Parallel Trend Test

**Figure 2:**
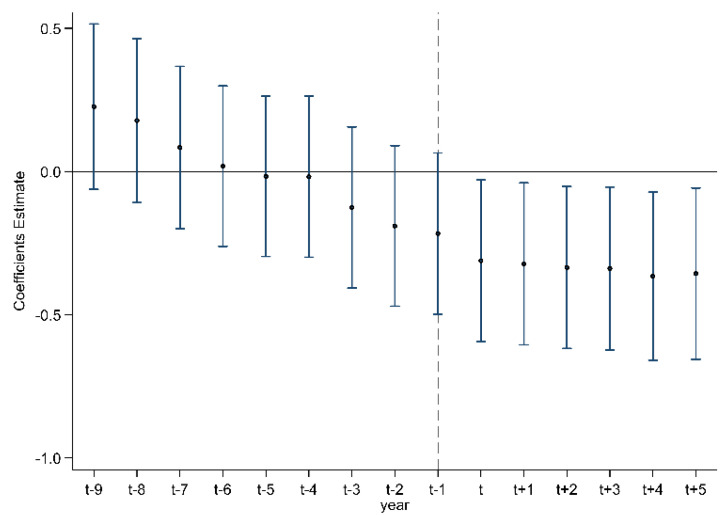
Parallel trend.

This paper uses the difference-in-difference method to study the impact of environmental information announcements on firm pollution emissions. An important requirement for this method is that the treatment group and the control group must have similar time trends before the policy is implemented, and there will be obvious differences after the policy is implemented. Therefore, before the empirical analysis, there should be a parallel trend test, which is conducted by the method of regression in this paper, and the regression results are shown in Table 2. Column (1) in Table 2 lists the clustered regression results at the prefecture-level city level fixed effects of the enterprises and the year without adding the control variables. The regression results before 2007 were not significant, while the regression results in 2008–2012 were significantly negative, which can indicate that the impact of environmental information disclosure on pollution emissions meets the parallel trend hypothesis. The clustered regression results at the prefecture-level city level following the further addition of control variables are shown in column (2) of Table 2, and what is different from column (1) is that after the adding of control variables, the environmental information announcements begin to show a significant impact on enterprises pollution emissions since 2007, which represents that the regression results in Table 2 basically meet the parallel trend hypothesis considering that the year, when the environmental information announcement system began to be implemented, was 2007. Figure 2 is the corresponding parallel trend graph, where the upper and lower dashed lines represent 95% confidence interval.

### 4.2. Firm Pollution Emissions Effect of Environmental Information Announcement

Table 3 presents the test results of firm pollution emissions effect of environmental information announcement, of which columns (1) and (2) are the regression results of all prefecture-level cities showing that the regression coefficient of environmental information announcement is significantly negative at the level of 1%, that is, the implementation of environmental information disclosure system in cities plays a negative role in curbing firm pollution emissions. As for the corresponding control variables, the impact of company size on firm pollution emissions is significantly positive, which means that the larger the company, the more pollution the company emits. The impact of company age on firm pollution emissions is significantly positive, possibly attributed by the reason that the long-lasting companies often have more resources to carry out production activities and emit more pollution. The impact of company’s main business income on firm pollution emissions is also significantly positive, the possible reason of which is that a high main business income implies a higher output that results higher pollution emission.

However, considering that the cities implementing the environmental information announcement system are widely distributed in the eastern, central, and western regions, and the cities are different, there are also great differences in the factor endowments between them. Therefore, we assume that these various factors lead to large differences between the cities in the control group and the control group, which may cause some biases in the results. Therefore, to further reduce the differences between samples and make the regression results more accurate, this paper adopts the propensity score matching method (PSM) to select the control group. Specifically, this paper performs period-by-period matching on the cross-section of the panel data to reduce the selection error of the control group. After excluding cities with large differences, DID regression is performed, and the results are shown in columns (3)–(4) of Table 3. It can be observed that regardless of whether or not adding control variables, the coefficient of environmental information announcement is significantly negative at the 1% level, which is consistent with the regression results before merging, which further indicates that environmental information announcement can effectively reduce firm pollutant emissions.

The possible reasons are as follows: First, the central government’s assessment mechanism for local officials has gradually been improved. It no longer uses GDP alone as an indicator, but more considers environmental protection, and uses environmental pollution as an indicator of the assessment system to prevent local officials from only paying attention to short-term interests, one-sided economic development, high investment and high pollution in exchange for economic development. In addition, the superior governments will not only directly supervise and inspect the environmental protection of local officials during their term of office, but also extensively listen to the opinions of the public and the media on the ecological environment and pollution emissions. This means that serious environmental pollution problems in cities will absolutely damage the performance evaluations of local officials. The “files” also clearly pointed out that local environmental protection departments should give full play to their duties. In addition to publicize environmental laws and other policy documents to the public, they are also required to take the initiative to disclose the local environmental quality status, the total discharge of major pollutants, implementation status of documents, as well as announcement documents for administrative penalties of enterprises violating environmental laws, so that the public understand the environmental situation in the region and the government environmental protection enforcement efforts. As the result of all changes above, local governments of the 113 environmental protection cities that the state focus on in the “files” will certainly pay more attention to local environmental issues, strengthen restrictions and regulations on local enterprises, and effectively reduce corporate pollution emissions. Second, according to the theory of polycentric environmental governance, increasing public participation can improve the efficiency of national resource allocation and create social benefits. Giving the public more rights to participate in environmental governance decision-making and increasing the public’s enthusiasm for participating in environmental governance can more effectively supervise enterprise production behavior and reduce pollutant emissions. Environmental information announcement can effectively alleviate the problem of information asymmetry between the government and the public. So, the active announcement of the discharge and treatment of major pollutants made by local governments and enterprises is able to establish communication channels and trust mechanisms with the public. Simultaneously, local governments usually cannot continuously and effectively supervise the emission reduction behavior of enterprises due to information asymmetry between the government and enterprises, while in the case of environmental information announcement, the public, to some extent, can be used as a substitute or supplement of local environmental protection departments, carrying out normal supervision of polluting enterprises and urging them to switch to green production methods to reduce pollution emissions.

### 4.3. Instrumental Variable Test

On the one hand, the choice of cities implementing environmental information announcement may be inseparable from the quality of the local environment. That is, cities with poor urban environments are often more likely to be selected as pilot cities for information announcement. Therefore, the mutual causality can conduct endogenous problems of environmental information announcement and firm pollution emissions. On the other hand, the emission of firm pollution will also be affected by the preferences of corporate managers and the policy environment, which are difficult to obtain fairly. Taking these two factors into consideration, our study is likely to have endogenous problems caused by two-way causality or ignoring important control variables. Therefore, we further adopt the number of types of newspapers issued by each city as an instrumental variable for environmental information announcement, for reasons as follows: First, although the internet has become the most widely available and easily accessible information transmission tool nowadays, considering that the research sample in this article is from 2004 to 2012, the most common media tools at that time were newspapers and television stations. In addition, local officials tend to pay more attention to local newspapers and learn about local people’s livelihood aspirations. When environmental issues are frequently mentioned in the newspapers, they will encourage local environmental disclosure systems. The more types of local newspapers, the greater the capacity for information collection and exposure, and the greater the probability that the city’s environmental information will be disclosed. The relationship indicates that our instrument meets the requirements of being correlative to the independent variable. Second, the number of types of newspapers, which is closely related to the local economic level and media development, will not change significantly with the change of firm pollution emissions, meeting the instrument requirements of being independent of the dependent variable. In summary, the types and quantities of newspapers, which we choose to make the instrumental variables for environmental information announcement, satisfy the basic assumption of instrumental variables. In this paper, the effect of environmental information announcement on firm pollution emissions is re-examined by the two-stage instrumental variable method (2SLS). The specific regression results are shown in Table 4.

Column (1) in Table 4 presents the impact of environmental information announcement on firm pollution emissions only, and column (2) presents this impact after the inclusion of control variables. It is shown that the coefficient estimates of the number of newspaper types is positive and significant at the 1% level. Basically, our proposed instrument satisfies the relevance of the IV approach. Furthermore, according to the empirical criteria, it appears that there is no weak instrument problem based on the fact that the F-statistic is much larger than 10. After removing endogeneity of environmental information announcement, the estimated coefficient on environmental information announcement is still negative significantly at the 1% level, and the coefficient size is 0.482 when control variables are included, which much larger than the 0.1852 of baseline regression. This further suggests that the environment information announcement can effectively reduce firm pollution emissions.

### 4.4. Test of Robustness

Although both the baseline regression and instrumental variable regression results indicate that environmental information announcement can effectively reduce firm pollution emissions, in order to ensure the credibility of the results, a robustness test will be performed by using alternative variables and changing the time span in this paper.

First, perform the robust test by changing the indicator of the independent variable. The pollutant emissions released by the company mainly include sulfur dioxide emissions, industrial wastewater emissions, and industrial dust emissions. Due to the considerable lack of industrial dust emissions in enterprises data, this paper chose industrial wastewater emissions to replace sulfur dioxide emissions as the indication of pollution emissions. In a similar way, we remove companies whose amount of industrial wastewater emissions is less than or equal to 0 and take the logarithmic processing. The specific regression results are shown in Table 5.

Columns (1)–(2) in Table 5 are shown the logarithms of industrial wastewater emissions that characterize firm pollution emissions. In the case of fixing firm and year effects, the coefficients on the environmental information announcement are significantly negative whether or not to add control variables, indicating that the replacement of company pollution emission indicator does not affect the inhibitory effect of environmental information announcement on the of environmental pollution emissions. By this result, the reliability of the results of this article gets furtherly demonstrated.

Second, this paper carries out the robustness test by changing the time span. In order to implement the strategy of sustainable development and prevent adverse effects on the environment after planning or implementing the project process, The Environmental Impact Assessment Law of the People’s Republic of China was officially promulgated in September 2003. This law, encouraging appropriate participation in environmental impact assessments by relevant units, experts and the public, has a certain relevance to the environmental information announcement system that was formally implemented in 2008. Taking the law in consideration, this article will adopt the 2003–2012 Chinese industrial enterprise panel data for the robustness test. Columns (3)–(4) of Table 5 provide the corresponding regression result. It can be concluded that in the case of fixing enterprise and year effects, the coefficients of environment information announcement are significantly negative whether or not to add control variables, indicating that the change of time span does not affect the inhibitory effect of environmental information announcement on environmental pollution emissions, and thus, further demonstrating the reliability of the results of this article.

## 5. Heterogeneity Test

### 5.1. Regional Heterogeneity

In a country as large as China, local characteristics, including the effect of environmental information announcement, differ from region to region. Therefore, dividing China into the eastern and central and western regions, this paper empirically studies the effect of environmental information announcement on firm pollution emissions. The specific regression results are shown in Table 6.

Columns (1)–(2) report the impact of environmental information announcement in the eastern region on firm pollution emissions. The result suggests that the estimated coefficient on environmental information announcement in the eastern region is significantly negative at the 1% level irrespective of control variables. Columns (3)–(4) report the impact of environmental information announcement in the central and western regions on firm pollution emissions, suggesting that the estimated coefficient on environmental information announcement in the central and western regions is not significant. The possible reasons are as follows: First of all, due to the relatively high level of economic development of eastern regions, local governments are able to pay more attention to high-quality economic growth rather than traditional GDP growth, resulting in that more attention is attached to environmental protection issues and more supervision implemented to polluting companies in eastern regions. Secondly, compared with the central and western regions, eastern regions have more high-tech enterprises and innovative enterprises, which produce clean energy. In addition, advanced production methods and rich management experience, which seem to be features of eastern regions, will also effectively improve the efficiency of energy use, thereby curbing pollution emissions. Finally, the public in eastern regions has more access to environmental announcement information thanks to the higher Internet penetration rate and has stronger demand for improving environmental quality, so they are more inclined to participate in social governance and supervise polluting companies, which in turn encourages polluting companies to reduce emissions. In addition, according to the pollution refuge theory, some polluting companies move to the central and western regions because of the weak supervision there, which is also one of the reasons why the environmental information announcement system in the central and western regions is not effective.

### 5.2. Urban Hierarchy

Furthermore, all prefecture-level cities are divided into first-class and second-class cities according to city levels for regression. The specific regression results are shown in Table 7.

Columns (1)–(2) report the impact of environmental information disclosure in first-class cities on firm pollution emissions, suggesting that when control firm fixed effects and year fixed effects, the coefficient of the first type of urban environmental information announcement is significantly negative at the 1% level, irrespective of adding control variables. Columns (3)–(4) report the impact of environmental information announcement in second-class cities on firm pollution emissions when fixing firm and year effects. The result suggests that the estimated coefficients on environmental information announcement in second-class cities are not significant and are much smaller than that of the first-class cities, which means that the environmental information announcement system in the second-class cities does not have a significant impact on firm pollution emissions.

### 5.3. Regulation Intensity

Although the environmental information announcement system has been implemented in all 113 prefecture-level cities, the stringency of implementation varies from city to city, based on which, emission reduction effects of prefecture-level cities may also be different. Therefore, according to the PITI index scores disclosed in 2008, this paper divides 113 cities in the treatment group into three levels of high regulatory intensity, medium regulatory intensity, and low regulatory intensity in detail, and studies the effect of environmental information announcement system on firm pollution under different regulatory intensities. The specific regression results are shown in Table 8. 

Columns (1)–(2) report the firm pollution emission impact of environmental information announcement under high regulatory intensity, the coefficients of which are significantly negative at the 1% level. Columns (3)–(4) report the firm pollution emission impact of environmental information announcement under medium regulatory intensity, the coefficients of which is significantly negative at the 1% level, and are lower compared with that of high regulatory intensity. Columns (5)–(6) report the impact of environmental information announcement on firm pollution emissions under low regulatory intensity, the coefficients of which are significantly positive at the 1% level. Thus, it can be concluded that the stringency of regulation plays a vital role in the effectiveness of the environmental information announcement system. The higher the regulation stringency, the better the corporate emission reduction effect of the environmental information announcement system. Additionally, even under the low regulatory intensity, the environmental information announcement system will promote the increase of firm pollution emissions. The regression results in this section are consistent with what Bao et al. (2013) have mentioned: if law enforcement is not strict, environmental legislation is difficult to obtain effective control effect, and even generates negative effects [25].

## 6. Transmission Mechanism

The foregoing empirical results indicate that environmental information announcements can significantly inhibit firm pollution emissions. Now, the question is how does it achieve it? The mediation effect model, including three regression equations, was established to explore the mechanism of urban environmental information announcement.

(2) $$Z_{it}=\delta_{0}+\beta_{1}\left(treat_{i}\times post_{t}\right)+\beta_{2}M_{it}+\vartheta_{i}+\mu_{i}+\varepsilon_{it}$$

(3) $$D_{it}=\delta_{0}+\theta_{1}\left(treat_{i}\times post_{t}\right)+\theta_{2}M_{it}+\vartheta_{i}+\mu_{i}+\varepsilon_{it}\;$$

(4) $$Z_{it}=\delta_{0}+\rho_{1}\left(treat_{i}\times post_{t}\right)+\rho_{2}M_{it}+\rho_{3}D_{it}+\vartheta_{i}+\mu_{i}+\varepsilon_{it}$$

Where the main dependent variable of interest is $Z_{it}$, measured by the emission of SO_2_ emitted by enterprises. $treat_{it}$ is the city grouping variable and takes 1 if city i implements the environmental information announcement system and takes 0 otherwise; $post_{it}$ is the time grouping variable, equaling 1 for years after 2008 and equaling 0 otherwise; $M_{it}$ is the set of control variables. In Formula (3), the mediating variable $D_{it}$ is the technological innovation (*lninn*), the output (*lnout*) and the end-of-end environmental governance(*lngov*), respectively, represented by the logarithm of the number of invention patents obtained by the company in the year, the logarithm of the company’s total output value of the year and the SO_2_ removal rate of the company which is characterized by (SO_2_ production-SO_2_ emissions)/SO_2_ production. $\beta_{i}$ is the estimated coefficient of each explanatory variable, $\delta_{0}$ is the intercept term, $\vartheta_{i}$ is the time-fixed effect, $\mu_{i}$ is the firm-fixed effect, $\varepsilon_{it}$ is the random error term of the econometric model. According to the definition of mediation effect, if $\beta_{1}$, $\theta_{1}$ and $\rho_{3}$ are significant, and $\rho_{1}$ is less than $\beta_{1}$ or significantly decrease, then the existence of mediation effect is proved. The regression results of specific mediating effects are shown in Table 9.

The impact of the environmental announcement on firm pollution emissions by not joining the intermediate variable are shown in column (1) of Table 9, which is consistent with the results in column (2) in Table 3, indicating that environmental information announcement can significantly inhibit firm pollution emissions. By analyzing the result reported by columns (1)–(3) in Table 9, it is possible to explore whether environmental information announcements can reduce pollution emissions through the mechanism of promoting enterprise technological innovation. The estimated coefficients on environmental information disclosure in column (2) is significantly positive at the 1% level, indicating that environmental information announcement can significantly promote enterprises to carry out technological innovation. In addition, the estimated coefficients on environmental information announcement in column (3) is significantly negative at the 1% level, and the smaller coefficient 0.0536 compared with the 0.1852 in column (1) indicates that technological innovation meets the criterion of mediating variables, that is, under the environmental information announcement system, companies will reduce pollution emissions through technological innovation. Environmental information announcement requires companies to voluntarily disclose the total annual resource consumption, environmental protection investment and total technological development. Additionally, specific polluting enterprises are also required to disclose the patterns and quantities of major pollutant emissions. With the increasing income levels inducing higher environmental demands of the public, the announcement of environmental information of polluting companies will confront supervision and constraints from both the government and the public. Under the pressure of social exposure and the promotion of public opinions, enterprises are tended to actively carry out technological innovation, accelerate to change the mode of production and promote green production to reduce pollutant emissions.

From columns (1) and (4)–(5) in Table 9, it is possible to explore whether environmental information announcement inhibits enterprises emitting through the mechanism of reducing production. In column (4), the estimated coefficients of environmental information announcement is significantly negative at the 1% level, indicating that environmental information announcement will suppress enterprises’ output. In addition, the coefficients on environmental information announcement in column (5) is significantly negative at the 1% level, and the smaller coefficient 0.1757 compared with the 0.1852 in column (1) indicates that enterprises’ output meets the criterion of mediating variables, that is, under the environmental information announcement system, companies choose to achieve the goal of reducing pollution emissions by reducing production. Confronted with the pressure of public opinions and public supervision brought about by environmental information announcements, some capable companies choose to carry out green production by technological innovation and environmentally friendly methods, but there are also many companies that do not have the capital and technology to transform their production methods. For these incapable enterprises, they have no choice but to adjust their production plans and sacrifice part of their output value to reduce pollution emissions and deal with external pressure from the government and the public. In this way, the company’s goal of reducing emissions seems to have been achieved in the short term, but it is tantamount to drinking poison to quench thirst in the long run and is not conducive to the long-term development of the company. In addition, once the policy supervision is negligent or the mass supervision is not in place, companies are likely to make a comeback and continue to emit large amounts of pollutants.

From columns (1), (6) and (7) in Table 9, it is possible to explore whether environmental information announcement inhibits enterprises emitting pollution through the mechanism of end-of-end environmental governance. In column (6), the estimated coefficients of environmental information announcement are significantly positive at the level of 1%, indicating that environmental information announcement will promote the end-of-end environmental governance of pollutants by enterprises. In addition, the estimated coefficients of environmental information disclosure in column (7) are significantly negative at the 1% level, and the smaller coefficient 0.1687 compared with 0.1852 in column (1) indicates that end-of-end environmental governance meets the criterion of mediating variables, that is, under the environmental information announcement system, companies will achieve the goal of reducing pollution emissions through end-of-line governance. The end-of-end environmental governance mainly refers to the removal of pollutants generated during the production process of the enterprise by means of the installation of pollution treatment equipment. Compared with technological innovation, which requires a large investment of manpower and material resources and does not necessarily obtain a stable income, it is undoubtedly a more effective way of end-of-end environmental governance. Because environmental information disclosure requires enterprises to publish the types and quantities of pollutants they emit, excessive pollution emissions are bound to cause concern to the government and the public, companies will deal with pollutants through waste gas pollution treatment facilities and desulfurization treatment facilities to reduce the amount and concentration of pollutant emissions.

Environmental information disclosure is the social responsibility of enterprises, which can play the role of social supervision and promote the construction of a modern environmental governance system. Based on the test of the transmission mechanism in this section, it can be concluded that under the environmental information announcement system, there is a dual mechanism of internal drive and external pressure in companies. On the one hand, under the supervision of the public, enterprises will actively carry out technological innovation or pollution end treatment, accelerate the transformation of production methods, promote green production, and reduce pollutant emissions. On the other hand, driven by public opinion, enterprises with small assets do not have enough capital and technology to change their production methods, and can only reduce pollutant emissions by sacrificing part of their output value. Therefore, to deal with environmental information announcement, the internal requirements drive companies to carry out front-end technological innovation and end-of-end environmental governance, while, under external pressure, enterprises achieve the goal of reducing pollution emissions by reducing production. Under the current background of "carbon peak" and "carbon neutrality", we should pay more attention to the role of technological innovation and end-of-life governance in promoting low-carbon economic development, introduce preferential subsidies to reduce the costs and risks of enterprise innovation and clean governance, and give full play to the due meaning of environmental information disclosure policy.

## 7. Conclusions

As the deterioration of the ecological environment has become an important factor restricting the high-quality development of China’s economy, the report of the 19th National Congress of the Communist Party of China clearly stated that environmental governance must be strengthened, and the development concept that lucid waters and lush mountains are invaluable assets should be established and practiced. To deal with the increasingly intensified contradiction between China’s environmental pollution and economic development, the Chinese government has implemented a series of environmental protection legislation and corresponding policies to promote the sustainable development of the ecological environment. So far, there has been abundant literature researching and evaluating this series of environmental regulations and policies implemented in China, but few have studied the impact of environmental information announcements on firm pollution emissions. On the one hand, this is partly because of the fact that the flow of information is not easily accessible to be observed and identified; On the other hand, the endogenous problems caused by the flow of information that affects the final research also increase difficulty in research. The “Measures for the Announcement of Environmental Information (for Trial Implementation)” policy officially implemented by the central government in 2008, as a "natural" exogenous shock, provides excellent conditions for this article to identify the impact of environmental information announcement on pollution emissions. This paper uses the exogenous impact of the environmental information announcement system as a quasi-natural experiment and merges China’s Industrial Enterprise Pollution Database with China’s Industrial Enterprise Database to obtain the panel data of industrial enterprises from 1998 to 2012. Then, we use the difference-in-difference model to test the effect of environmental information announcements on firm pollution emissions. The main conclusions are as follows: (1) The environmental information disclosure system has significantly suppressed corporate pollution emissions. The empirical results suggest that the pollution emissions of industrial enterprises in cities that implemented the environmental information announcement system have dropped significantly compared with cities that do not publish environmental information. (2) The impact of the environmental information announcement system on firm pollution emissions varies according to the region, city level, and environmental regulation intensity. Specifically, first, the environmental information announcement system in the eastern region can effectively promote the emission reduction of industrial enterprises, while it has no significant impact in the central and western regions. Second, the environmental announcement system in the first-class cities can significantly restrain the problem of firm pollution emissions, while the impact in the second-class cities is not significant. This is mainly because the former are easier to develop green technologies due to their economic, talent, and technological advantages, and at the same time, the public there are inclined to participate in the supervision of government inactions and enterprise’ polluting behavior. Third, the implementation of an environmental information announcement system in regions with high and medium regulatory intensity can significantly inhibit corporate pollution emissions, and with the increase in regulatory intensity, the suppression effect will become more significant. However, in regions with low regulatory intensity, the system will instead increase corporate pollution emissions. (3) Under the environmental information announcement system, companies reduce pollution emissions through the mechanism of internal drive of front-end technological innovation and end-end environmental governance; in addition, under external pressure, companies can achieve the goal of reducing pollution emissions by reducing output.

The research results of this paper have the following policy implications: First, local governments should continuously strengthen and improve the environmental information announcement system to solve the problem of information asymmetry between local governments and the public, so that the public can understand and participate in environmental governance to further improve environmental governance system. Second, to build a communication platform based on the announcement of environmental regulatory information, local governments should not only let the public know about environmental issues, but also improve the public’s ability to participate in the environment. Encourage multiple entities such as the government, enterprises and the public to participate in urban environmental governance and promote the construction of the interaction of environmental protection behavior and the mutual trust system in environmental policies. Third, build a differentiated policy intervention system. The empirical results suggest that in cities with different regions, different levels of cities, and different environmental regulatory intensities, the market-based policy intervention system presents obviously differentiated results. Therefore, it is advisable to formulate and construct differentiated policies combining city characteristics, and scientifically assess local governments’ environmental governance performance under different government systems.

There are a few limitations in this study. Due to the availability and comprehensiveness of data, the sample range of this paper is up to 2012. In addition, with the development of information technology, the ways and means of environmental information disclosure are gradually increasing. The current study only evaluates the effect of the measures for environmental information disclosure (for Trial Implementation) but would not represent the carbon emission reduction effect of all environmental information disclosure. In the future, on the one hand, through the collection and investigation of relevant data, we can explore the radiation effect or siphon effect of policies from a spatial perspective, so as to provide more empirical evidence for the impact of environmental information disclosure on carbon emission reduction of enterprises. On the other hand, in order to evaluate the effects of policies more comprehensively, we can compare the effects of different environmental information disclosure methods on carbon emission reduction.

## Figures and Tables

**Table 1:** Variable descriptive statistics.

*Symbol*	*Variable Name*	*Obs*	*Mean*	*Std. Dev.*	*Min*	*Max*
*lnSO_2_*	*SO_2_ emission*	443,777	9.763	2.112	0.693	18.192
*PITI*	*environmental information disclosure*	443,778	0.216	0.412	0	1
*lnsca*	*Enterprise scale*	443,735	10.836	1.646	0	19.358
*lnage*	*Enterprise age*	443,378	2.369	0.892	0	7.607
*lninc*	*Enterprise revenue*	443,687	10.865	1.700	0	19.054
*soe*	*Ownership type*	443,778	0.203	0.402	0	1

**Table 2:** Parallel trend test.

*Variables*	(1)	(2)
	*lnSO_2_*	*lnSO_2_*
*t-9*	0.2314	0.2264
	(0.1481)	(0.1473)
*t-8*	0.1859	0.1778
	(0.1469)	(0.1462)
*t-7*	0.0980	0.0838
	(0.1452)	(0.1445)
*t-6*	0.0314	0.0187
	(0.1436)	(0.1429)
*t-5*	0.0014	−0.0171
	(0.1435)	(0.1427)
*t-4*	0.0022	−0.0181
	(0.1444)	(0.1436)
*t-3*	−0.1056	−0.1255
	(0.1443)	(0.1435)
*t-2*	−0.1688	−0.1902
	(0.1437)	(0.1430)
*t-1*	−0.2008	−0.2172
	(0.1446)	(0.1438)
*t*	−0.3054 **	−0.3118 **
	(0.1446)	(0.1439)
*t+1*	−0.3233 **	−0.3226 **
	(0.1450)	(0.1443)
*t+2*	−0.3442 **	−0.3353 **
	(0.1450)	(0.1443)
*t+3*	−0.3478 **	−0.3389 **
	(0.1459)	(0.1452)
*t+4*	−0.3928 ***	−0.3657 **
	(0.1504)	(0.1498)
*t+5*	−0.3905 **	−0.3568 **
	(0.1535)	(0.1527)
*lnsca*		0.0313 ***
		(0.0054)
*lnage*		0.0639 ***
		(0.0056)
*lninc*		0.1254 ***
		(0.0044)
*soe*		0.0286 **
		(0.0137)
*_cons*	9.9145 ***	8.0501 ***
	(0.0837)	(0.1029)
*Firm fixed effect*	*YES*	*YES*
*Year fixed effect*	*YES*	*YES*
*N*	414,112	413,626
*R^2^*	0.7561	0.7587

Notes: ***, ** denote the significance levels of 1%, 5%, respectively.

**Table 3:** Enterprises pollution emissions effect of environmental information announcement.

*Variables*	(1)	(2)	(3)	(4)
	*lnSO_2_*	*lnSO_2_*	*lnSO_2_*	*lnSO_2_*
*PITI*	−0.2130 ***	−0.1852 ***	−0.1492 ***	−0.1309 ***
	(0.0251)	(0.0248)	(0.0273)	(0.0269)
*lnsca*		0.0309 ***		0.0389 ***
		(0.0054)		(0.0077)
*lnage*		0.0610 ***		0.0594 ***
		(0.0057)		(0.0081)
*lninc*		0.1268 ***		0.1361 ***
		(0.0044)		(0.0061)
*soe*		0.0112		0.0390 **
		(0.0139)		(0.0193)
*_cons*	9.8663 ***	7.9924 ***	10.0502 ***	7.9849 ***
	(0.0066)	(0.0602)	(0.0065)	(0.0844)
*Firm fixed effect*	YES	YES	YES	YES
*Year fixed effect*	YES	YES	YES	YES
*N*	414,112	413,626	195,005	195,005
*R^2^*	0.7555	0.7581	0.7705	0.7733

Notes: ***, ** denote the significance levels of 1%, 5%, respectively.

**Table 4:** Test of Instrumental Variables.

*Variables*	(1)	(2)	(3)	(4)
	*First stage*	*Second stage*	*First stage*	*Second stage*
	*PITI*	*lnSO_2_*	*PITI*	*lnSO_2_*
*IV*	0.046 ***		0.046 ***	
	(0.000)		(0.000)	
*PITI*		−0.554 ***		−0.482 ***
		(0.036)		(0.036)
*lnsize*			0.001	0.028 ***
			(0.001)	(0.005)
*lnage*			−0.001	0.060 ***
			(0.001)	(0.005)
*lninc*			−0.008 ***	0.122 ***
			(0.001)	(0.004)
*Soe*			0.015	0.020
			(0.002)	(0.014)
*Firm fixed effect*	*YES*	*YES*	*YES*	*YES*
*Year fixed effect*	*YES*	*YES*	*YES*	*YES*
*N*	363,500	363,500	363,110	363,110
*R^2^*		0.002		0.013
*First stage F-Value*	14,417.67		14,285.59	
*Kleibergen-Paap rk LM statistic*		21,000 *** (0.000)		21,000 *** (0.000)
*Cragg-Donald Wald F statistic*		57,000		56,000
*Kleibergen-Paap rk Wald F statistic*		14,000		14,000

Notes: *** denotes the significance levels of 1%.

**Table 5:** Robust test.

*Variables*	(1)	(2)	(3)	(4)
	*lnwater*	*lnwater*	*lnSO_2_*	*lnSO_2_*
*PITI*	−0.1228 ***	−0.0884 ***		
	(0.0243)	(0.0239)		
*PITI2003*			−0.1526 ***	−0.1236 ***
			(0.0252)	(0.0248)
*lnsca*		0.0617 ***		0.0497 ***
		(0.0066)		(0.0067)
*lnage*		0.0764 ***		0.0630 ***
		(0.0060)		(0.0075)
*lninc*		0.1233 ***		0.1191 ***
		(0.0051)		(0.0056)
*soe*		0.0494 ***		0.0202
		(0.0139)		(0.0201)
*_cons*	10.7124 ***	8.4863 ***	9.8195 ***	7.7831 ***
	(0.0065)	(0.0766)	(0.0089)	(0.0831)
*Firm fixed effect*	*YES*	*YES*	*YES*	*YES*
*Year fixed effect*	*YES*	*YES*	*YES*	*YES*
*N*	327,063	326,728	298,253	298,040
*R^2^*	0.8016	0.8044	0.7759	0.7778

Notes: *** denotes the significance levels of 1%.

**Table 6:** The test of regional heterogeneity.

*Variables*	(1)	(2)	(3)	(4)
	*lnSO_2_*	*lnSO_2_*	*lnSO_2_*	*lnSO_2_*
*PITI ×* *east*	−0.1645 ***	−0.1324 ***		
	(0.0303)	(0.0298)		
*PITI ×* *middle*			−0.0040	−0.0245
			(0.0375)	(0.0369)
*lnsca*		0.0404 ***		0.0408 ***
		(0.0078)		(0.0079)
*lnage*		0.0600 ***		0.0589 ***
		(0.0084)		(0.0084)
*lninc*		0.1341 ***		0.1361 ***
		(0.0063)		(0.0063)
*soe*		0.0451 **		0.0424 **
		(0.0198)		(0.0199)
*_cons*	10.0272 ***	7.9660 ***	10.0052 ***	7.9254 ***
	(0.0057)	(0.0862)	(0.0048)	(0.0869)
*Firm fixed effect*	*YES*	*YES*	*YES*	*YES*
*Year fixed effect*	*YES*	*YES*	*YES*	*YES*
*N*	187,234	187,234	187,234	187,234
*R^2^*	0.7728	0.7755	0.7726	0.7754

Notes: ***, ** denote the significance levels of 1%, 5%, respectively.

**Table 7:** The test of urban hierarchy heterogeneity.

*Variables*	(1)	(2)	(3)	(4)
	*First-Class Cities*		*Second-Class Cities*	
	*lnSO_2_*	*lnSO_2_*	*lnSO_2_*	*lnSO_2_*
*PITI × first*	−0.2700 ***	−0.2371 ***		
	(0.0395)	(0.0383)		
*PITI × second*			−0.0377	−0.0248
			(0.0307)	(0.0301)
*lnsca*		0.0311 ***		0.0321 ***
		(0.0056)		(0.0056)
*lnage*		0.0613 ***		0.0622 ***
		(0.0058)		(0.0058)
*lninc*		0.1279 ***		0.1292 ***
		(0.0045)		(0.0046)
*soe*		0.0154		0.0111
		(0.0142)		(0.0143)
*_cons*	9.8214 ***	7.9361 ***	9.8103 ***	7.8984 ***
	(0.0050)	(0.0623)	(0.0056)	(0.0626)
*Firm fixed effect*	YES	YES	YES	YES
*Year fixed effect*	YES	YES	YES	YES
*N*	400,501	400,074	400,501	400,074
*R^2^*	0.7568	0.7594	0.7565	0.7591

Notes. *** denotes the significance levels of 1%.

**Table 8:** The test of regulation intensity heterogeneity.

*Variables*	(1)	(2)	(3)	(4)	(5)	(6)
	*High*		*Medium*		*Low*	
	*lnSO_2_*	*lnSO_2_*	*lnSO_2_*	*lnSO_2_*	*lnSO_2_*	*lnSO_2_*
*PITI ×* *One*	−0.1465 ***	−0.1109 ***				
	(0.0336)	(0.0329)				
*PITI ×* *Two*			−0.1464 ***	−0.1443 ***		
			(0.0345)	(0.0335)		
*PITI ×* *Three*					0.0408	0.0309
					(0.0372)	(0.0369)
*lnsca*		0.0325 ***		0.0315 ***		0.0321 ***
		(0.0056)		(0.0056)		(0.0056)
*lnage*		0.0621 ***		0.0621 ***		0.0623 ***
		(0.0058)		(0.0058)		(0.0058)
*lninc*		0.1275 ***		0.1296 ***		0.1293 ***
		(0.0045)		(0.0046)		(0.0046)
*soe*		0.0133		0.0123		0.0109
		(0.0142)		(0.0143)		(0.0143)
*_cons*	9.8203 ***	7.9209 ***	9.8175 ***	7.9089 ***	9.8038 ***	7.8932 ***
	(0.0052)	(0.0623)	(0.0054)	(0.0627)	(0.0046)	(0.0628)
*Firm fixed effect*	YES	YES	YES	YES	YES	YES
*Year fixed effect*	YES	YES	YES	YES	YES	YES
*N*	400,501	400,074	400,501	400,074	400,501	400,074
*R^2^*	0.7566	0.7592	0.7566	0.7592	0.7565	0.7591

Notes. *** denotes the significance levels of 1%.

**Table 9:** Transmission mechanism.

*Variables*	(1)	(2)	(3)	(4)	(5)	(6)	(7)
	*lnSO_2_*	*lninn*	*lnSO_2_*	*lnout*	*lnSO_2_*	*lngov*	*lnSO_2_*
*lninn*			−0.0536 ***				
			(0.0121)				
*lnout*					0.0714***		
					(0.0058)		
*lngov*							−1.0825 ***
							(0.0214)
*PITI*	−0.1852 ***	0.0454 ***	−0.1868 ***	−0.0312 ***	−0.1757 ***	0.0153 ***	−0.1687 ***
	(0.0248)	(0.0039)	(0.0295)	(0.0049)	(0.0274)	(0.0049)	(0.0236)
*lnsca*	0.0309 ***	0.0133 ***	0.0313 ***	−0.0010	0.0306 ***	0.0063 ***	0.0377 ***
	(0.0054)	(0.0009)	(0.0058)	(0.0096)	(0.0057)	(0.0008)	(0.0054)
*lnage*	0.0610***	−0.0115 ***	0.0592 ***	0.0095 ***	0.0580 ***	0.0023 ***	0.0635 ***
	(0.0057)	(0.0011)	(0.0059)	(0.0023)	(0.0058)	(0.0009)	(0.0056)
*lninc*	0.1268 ***	0.0036 ***	0.1277 ***	0.8291 ***	0.0731 ***	0.0013 **	0.1283 ***
	(0.0044)	(0.0007)	(0.0047)	(0.0073)	(0.0062)	(0.0007)	(0.0044)
*soe*	0.0112	0.0172 ***	0.0112	−0.0078	0.0136	0.0030	0.0145
	(0.0139)	(0.0027)	(0.0143)	(0.0048)	(0.0143)	(0.0022)	(0.0136)
*_cons*	7.9924 ***	−0.1232 ***	8.0024 ***	1.9214 ***	7.8301 ***	0.0159 **	8.0096 ***
	(0.0602)	(0.0100)	(0.0633)	(0.1220)	(0.0667)	(0.0077)	(0.0608)
*Firm fixed effect*	*YES*	*YES*	*YES*	*YES*	*YES*	*YES*	*YES*
*Year fixed effect*	*YES*	*YES*	*YES*	*YES*	*YES*	*YES*	*YES*
*N*	413,626	379,931	379,930	377,021	377,020	413,610	413,610
*R^2^*	0.7581	0.4299	0.7537	0.9407	0.7576	0.4610	0.7656

Notes: ***, ** denote the significance levels of 1%, 5%, respectively. The numbers in parentheses are the standard deviations of the regression coefficients.

## Data Availability

The datasets used and analyzed in the current study are available from the corresponding author upon reasonable request.
